# Engineering the Immune–Stromal Microenvironment: A Design Framework for Regenerative Biomaterials

**DOI:** 10.3390/cells15191742

**Published:** 2026-09-24

**Authors:** Rezvan Mobasseri, Ali Ghiaseddin, Meraj H. Khan, Patrick L. Wagner, Neda Dadgar

**Affiliations:** 1 Department of Pharmacology, School of Medicine, Case Western Reserve University, Cleveland, OH 44106, USA; 2 Department of Chemistry, Michigan State University, East Lansing, MI 48824, USA; 3 Department of Cancer Sciences, Cleveland Clinic Research, Cleveland Clinic, 9500 Euclid Ave, Cleveland, OH 44195, USA; 4 Allegheny Health Network Cancer Institute, Pittsburgh, PA 15224, USA

**Keywords:** immunometabolism, immune regulation, extracellular vesicles, tissue microenvironment, macrophage–fibroblast crosstalk, foreign body response, biomaterial immune engineering

## Abstract

**Highlights:**

**What are the main findings?**

• Regenerative outcomes depend on how material-driven immune responses develop into stromal remodeling and tissue-level change.
• The four-layer framework shows where studies connect engineered inputs with biological, stromal, and functional outcomes and where those links remain untested.

**What is the implication of the main finding?**

• Biomaterial studies should move beyond isolated immune markers and include stromal, matrix, vascular, temporal, and functional measurements.
• The framework can guide more complete experimental assessment but requires prospective validation before predictive or clinical use.

**Abstract:**

The success of regenerative biomaterials depends not simply on biocompatibility but also on their ability to direct coordinated interactions between immune and stromal compartments that ultimately determine tissue repair, integration, or fibrosis. Rather than viewing inflammation and fibrosis as independent outcomes, this review presents a design framework that links engineered material properties to sequential immune activation, stromal remodeling, and long-term tissue function. We describe how material chemistry, architecture, mechanical properties, degradation behavior, surface modification, and spatiotemporal delivery of bioactive signals shape innate immune sensing, macrophage state transitions, adaptive immune regulation, and the macrophage–fibroblast axis. We further discuss how these interconnected processes regulate extracellular matrix remodeling, vascular adaptation, and fibrotic capsule formation, emphasizing that tissue fate emerges from the coordination of immune and stromal responses rather than from modulation of any single pathway. Drawing on this evidence, we propose a four-layer conceptual framework that organizes engineered inputs, biological processes, stromal responses, and tissue-level outcomes. Rather than functioning as a predictive model, the framework is intended as an evidence-mapping and experimental-design tool that makes explicit where causal links are directly supported, where they are inferred, and where they remain untested. We identify key translational gaps—including study heterogeneity, incomplete stromal and functional assessment, limited clinical validation, and insufficient longitudinal profiling—and propose practical priorities for multiparameter stromal analysis, high-dimensional myeloid phenotyping, intermediate mechanistic models, and application-relevant functional endpoints.

## 1. Introduction

The host response to biomaterials is no longer understood as a simple matter of tolerance versus rejection. Older ideas of biocompatibility often emphasized low toxicity, limited inflammation, or passive acceptance by the host [1,2]. That view remains useful, but it is incomplete. A material may look acceptable early after implantation and still fail later if inflammation does not resolve, fibroblasts remain activated, matrix accumulates, or the implant becomes isolated by a fibrotic capsule [3,4,5].

More recent work has shifted attention toward the sequence of events that connects the material design to the tissue outcome. The long-term fate of an implant or scaffold depends on coordinated changes across immune cells, fibroblasts, ECM, vasculature, local mechanics, and, in some settings, adaptive immune regulators [3,4,6]. The question is therefore not only whether inflammation is present but also whether the response is organized in a way that moves toward integration and remodeling rather than chronic inflammation and fibrosis [2,4,7].

The foreign body response (FBR) is one of the clearest examples of how implanted materials shape local immune–stromal organization [1,2,8]. As illustrated in Figure 1, the FBR unfolds through four temporally overlapping phases—protein adsorption, innate sensing and recruitment, macrophage activation and state transitions, and immune–stromal crosstalk—before diverging at a critical design decision point into either constructive remodeling and integration or chronic fibrosis and encapsulation. This sequential structure is not a fixed biological program. It is a series of designable transitions, each of which can be shifted by engineered material properties acting on the host response.

FBR should not be treated only as a pathological endpoint to eliminate. Several reviews argue that parts of the host response can be guided or harnessed when the goal is in situ tissue formation or controlled integration [4,7,9,10]. In tissue-engineered vascular grafts, Roh and colleagues showed that implanted grafts can transform into mature neo-vessels through an inflammation-mediated process of vascular remodeling [11]. Related in vivo tissue-engineering approaches also use host-driven peri-implant tissue formation to generate vascular graft-like structures, treating the foreign body capsule not only as a failure mode but also as a potential source of organized autologous tissue [10,12,13]. FBR is therefore not inherently beneficial or harmful; its outcome depends on how the host response is organized.

Constructive integration, vascularized repair, chronic inflammation, and fibrotic encapsulation can be understood as related outcomes of overlapping immune–stromal mechanisms rather than as fully separate biological programs [3,4,6,14,15]. This helps explain why strategies that target only one layer of the response may have limited effects: blocking a single cytokine or pushing macrophages toward one nominal phenotype may not be sufficient if inflammation, matrix remodeling, fibroblast activation, and vascular adaptation remain poorly coordinated [4,5,16,17,18].

This review develops a design-oriented framework for understanding how engineered tissue microenvironments shape tissue fate by translating early host sensing into stromal remodeling and long-term tissue outcomes. It begins with early host sensing and FBR biology, examines engineering inputs that shape innate activation, timing and state transitions, immune–stromal crosstalk, and the role of physical architecture and mechanics, and proposes a taxonomy that links engineered inputs to biological processes, stromal consequences, and tissue-level outcomes. The aim is to make the literature easier to use for biomaterial design, not to rank every study in the field. Existing reviews have described the stages of the foreign body response [1,2,8], the effects of biomaterial properties on immune responses [4,9], and macrophage–fibroblast interactions in repair and fibrosis [5,19]. Building on these perspectives, our four-layer framework asks whether a defined engineered input is linked within a study to a biological process, a stromal response, and a tissue-level outcome. It is intended to identify downstream links that remain untested, not to replace existing models or predict biomaterial performance.

## 2. Methodology

This review was conducted as a narrative concept-driven literature review rather than a systematic review or meta-analysis. The goal was not to exhaustively capture every study in the field but to develop a practical framework for understanding how engineered microenvironments shape tissue outcomes through linked immune and stromal processes.

Literature searches were conducted in PubMed and Web of Science, covering publications from 1999 to 2026. No language or publication type restrictions were applied during the initial search; non-English publications were excluded during screening. The search was complemented by the manual review of reference lists from highly cited studies in the biomaterial immune engineering field.

The guiding question was how engineered microenvironmental features influence innate immune sensing and how these early effects are carried forward into stromal remodeling and tissue-level outcomes. The literature was selected iteratively, with priority given to studies that linked a defined engineered variable to an immune pathway, immune-cell state transition, immune–stromal signaling relationship, stromal response, or tissue-level outcome.

Primary experimental studies were emphasized when they connected material chemistry, degradation behavior, surface modification, coating design, controlled release, geometry, porosity, topography, stiffness, or viscoelasticity to measurable biological and tissue responses. Translational studies were included when they connected these mechanisms to vascularization, extracellular matrix remodeling, fibrotic capsule formation, implant integration, functional repair, or device-relevant outcomes. Foundational studies and review articles were used selectively to provide historical context and broader conceptual framing.

Studies were given highest priority when they connected three elements: an engineered input, a biological mechanism, and a stromal or tissue-level outcome. Mechanisms of interest included protein adsorption, PRR-related sensing, myeloid recruitment, macrophage activation and state transitions, adaptive immune modulation, macrophage–fibroblast crosstalk, fibroblast organization, metabolic persistence, mechanotransductive signaling, and related pathways linking immune activity to stromal remodeling. Outcome domains included extracellular matrix organization, vascular adaptation, implant integration, fibrotic encapsulation, immune resolution or persistence, and functional tissue repair.

The selection criteria were conceptual rather than protocol driven. Studies were considered for detailed discussion when they examined a defined engineered variable together with at least one relevant immune, stromal, or tissue-level response. Studies were not prioritized when the engineered variable or the relevant biological outcome could not be identified clearly, while reviews were used mainly for conceptual context and reference tracing. Because this was a narrative review, no formal risk-of-bias instrument or quantitative study-quality score was applied. As with any narrative review, study selection involved author judgment and may not capture every relevant report.

## 3. Innate Immune Sensing as a Design Handle

At the earliest stage of host response, the central question is how the material is first sensed. Protein adsorption, tissue injury, material chemistry, degradation products, surface features, and local physical conditions all shape the first host–material interface [1,2,3,8]. PRR and inflammatory sensing pathways are part of that response, but in regenerative biomaterial settings they should be discussed in relation to material recognition, damage signals, and downstream repair or fibrosis rather than as a general immunology problem [2,3].

Figure 1 organizes this sequence into four phases that together constitute the FBR. In Phase 1, host proteins—fibrinogen, vitronectin, and fibronectin—deposit onto the material surface within minutes of implantation, forming a conditioned layer whose composition is determined by material chemistry and coating design. Classic experimental studies showed that adsorbed fibrinogen can drive acute inflammatory responses to implanted polymers, identifying protein adsorption as an early determinant of host recognition [20]. In Phase 2, PRR activation and release of damage-associated molecular patterns (DAMPs) drive neutrophil and monocyte infiltration, followed by macrophage arrival at the interface [1,3,8]. In Phase 3, macrophages adopt activation states spanning inflammatory (TNF-α, IL-1β, reactive oxygen species), reparative (IL-10, arginase-1), and pro-fibrotic (TGF-β, PDGF) programs, with metabolic state and physical material cues—shape, stiffness, topography—influencing which states predominate. In Phase 4, TGF-β and PDGF drive fibroblast recruitment, bidirectional macrophage–fibroblast signaling is established, adaptive immune inputs from Tregs, TH2 cells, and B cells modulate the niche, and ECM remodeling begins. The dashed conceptual Design Decision Window in Figure 1 represents a period during which the trajectory may remain modifiable: engineered inputs acting across the preceding phases can shift the response toward constructive remodeling and integration or toward chronic fibrosis and encapsulation.

Experimental studies confirm that this early sensing step is already design sensitive. In a combinatorial screen of chemically modified alginates, triazole-containing materials reduced macrophage recognition and recruitment and sharply limited fibrotic overgrowth after implantation, including reduced alpha-smooth muscle actin (αSMA)-positive stromal activation [21]. In another study, one-dimensional wrinkled topographies promoted macrophage elongation, increased arginase-1 and interleukin-10 (IL-10), reduced tumor necrosis factor-α (TNF-α), and altered collagen organization around implanted materials [22]. Early host recognition is therefore not fixed. It can be modulated by the initial design of the material.

Early sensing events also feed forward into stromal remodeling. In mechanically tunable polyethylene glycol (PEG) hydrogels spanning a fibrotic stiffness range, focal adhesion kinase (FAK)-dependent remodeling was accompanied by αSMA-positive myofibroblast activation, collagen I deposition, and fibrotic capsule formation. FAK inhibition reduced these stromal outcomes and altered the peri-implant immune niche [14]. Around nondegradable polymer implants, persistent glycolytic programming in implant-proximal macrophages was associated with continued fibroblast-driving inflammatory output and stabilization of a fibrotic niche [15]. The key issue is therefore not only whether innate immunity turns on, but how the intensity, duration, and location of that early response are translated into fibroblast behavior, matrix deposition, and either repair or fibrosis [5,6,19].

## 4. Engineering Inputs That Shape Innate Activation

One important strategy is to create localized material systems that recruit immune cells and expose them to immunomodulatory cues within a confined tissue environment. In these systems, the material does more than deliver a payload. It establishes a local immune niche.

Su and colleagues used scaffolds functionalized with exosomes isolated from bone-marrow-derived MSCs obtained from BALB/c mice. Local exosome retention promoted M2-like macrophage responses together with regulatory T-cell (Treg)- and T helper 2 (TH2)-associated immune activity, accompanied by improved collagen deposition, re-epithelialization, and vascularization in the wound bed [23]. Similar depot-based logic has been used for cytokine delivery in tissue repair. Sequential release of interferon-γ (IFN-γ) followed by interleukin-4 (IL-4) shaped macrophage functions in stages and supported later scaffold vascularization [16]. In another study, local IL-4 release from a biomimetic scaffold increased M2-associated markers and enriched cluster of differentiation 206 (CD206)-positive macrophages early after implantation [24], while an IL-4-eluting layer-by-layer coating on polypropylene mesh shifted the early macrophage phenotype at the implant interface and was associated with reduced capsule thickness and improved tissue integration at later time points [17].

The effects of MSC-derived EVs depend on the MSC source, culture and conditioning methods, EV isolation and characterization, dose, and storage. These factors can alter EV composition and potency and contribute to batch-to-batch variation. Limited local retention, clearance after delivery, and differences among injury sites further restrict comparison across applications. The current evidence is insufficient to determine whether scaffold-delivered MSC-EVs can reliably shift the Design Decision Window toward constructive remodeling in large-animal or clinical settings [25].

A second strategy relies less on released factors and more on the intrinsic properties of the material itself. The composition, charge, surface chemistry, degradation behavior, coating design, and interfacial interactions can all influence how strongly innate cells respond and what kind of response is produced [2,3,5].

In a volumetric muscle-loss model in female C57BL/6 mice, biologic ECM scaffolds induced type-2-associated immune programs and supported cellular infiltration, connective-tissue remodeling, and formation of new small muscle fibers. Compared with biologic ECM scaffolds, synthetic PE and PEG materials produced persistent neutrophil-rich responses and collagenous encapsulation, accompanied by peripheral αSMA immunostaining and higher deposition of mature collagen fibrils [26]. Vegas and colleagues showed that triazole-modified alginates reduced macrophage recruitment and limited fibrotic overgrowth in rodents and non-human primates [21].

The surface charge and coating thickness influenced fibrotic capsule formation around PDMS implants. Electronegative PAA/PEI coatings produced the lowest near-interface collagen density and the thinnest capsules after one month, whereas sufficiently thick coatings reduced the capsule thickness more effectively than nanometer- or low-micrometer-scale coatings [27]. The study did not directly characterize protein adsorption or immune-cell phenotype, leaving the biological mechanism linking coating properties to fibrosis unresolved. Together, these studies show that innate activation can be tuned not only by what a material delivers but also by what the material is and how its interface is presented to the host.

In biomaterial settings, macrophages are often described using classical M1/M2 language, but this shorthand does not fully capture how myeloid cells respond to material chemistry, geometry, stiffness, topography, degradation, and local tissue cues. The terms remain useful as broad labels for inflammatory or repair-associated programs, but they should not be treated as complete macrophage phenotypes. Macrophage states are dynamic, tissue-dependent, and shaped by both physical and biochemical context [5,18,28,29]. McWhorter and colleagues showed that enforcing macrophage elongation on micropatterned substrates increased M2-like markers even without exogenous cytokines, and the effect depended on actin organization and contractility [30]. From a design standpoint, the question is not simply whether a material produces an M2-like marker profile. The question is whether it supports the sequence of myeloid functions that the tissue needs: debris clearance, vascular support, matrix turnover, resolution, and control of fibrotic progression [18,29].

## 5. Timing, Persistence, and State Transitions

The timing of immune cues is often as important as their identity. In regenerative and implant settings, early inflammation is not automatically harmful. It can support immune-cell recruitment, debris clearance, angiogenic initiation, and conditioning of the local tissue environment [2,4,31]. Problems arise when this phase becomes prolonged, spatially diffuse, or poorly matched to later remodeling.

This is why sequential cytokine-delivery systems and locally controlled depots are useful examples. They are designed to align immune activation with the changing needs of tissue repair rather than expose the tissue to one static signal [2,4,9].

In vitro, short-term IFN-γ release followed by more sustained IL-4 presentation produced staged macrophage responses. In vivo, increased CD31-positive vascularization was most clearly observed in the IFN-γ-releasing condition, supporting the broader principle that cytokine timing can influence angiogenic remodeling [16]. Local IL-4 delivery provides a related design example: in a biomimetic collagen scaffold, IL-4 release enriched CD206-positive macrophages among cells infiltrating the scaffold in vivo at early time points [24], and an IL-4-eluting layer-by-layer coating was associated with reduced capsule formation and improved integration at 90 days [17].

Persistence also matters in chronic FBR. Around nondegradable polymer implants, implant-proximal macrophages showed persistent glycolytic programming well into chronic FBR, a metabolic state associated with continued fibroblast-driving inflammatory output and stabilization of a profibrotic niche [15]. Duration should not be considered only at the level of cytokine release. It also matters at the level of cellular metabolism and chronic immune-state maintenance. The design challenge is not to maximize or suppress inflammation in general. It is to control the sequence, duration, and localization of immune states so that inflammatory initiation can move into resolution and constructive remodeling [2,4,5,18]. The same immune cue can therefore support repair or promote pathology depending on when it appears, how long it persists, and whether it gives way to the next phase of remodeling.

## 6. From Innate Activation to Stromal Remodeling

If innate sensing is the stage at which biomaterials are first translated into a host response, stromal remodeling is where tissue fate begins to separate into repair or fibrosis. As shown in Figure 2, this transition is governed by the macrophage–fibroblast axis—a coupled system in which engineered material properties, adaptive immune signals, and mechanical cues all converge to determine whether remodeling resolves toward constructive integration or stabilizes into fibrotic encapsulation. Fibroblasts, ECM, vascular compartments, and local mechanics do not simply follow immune activity. They also shape immune-cell behavior and help determine whether the response resolves, remodels constructively, or progresses toward persistent fibrosis [5,6,19].

The left side of Figure 2 shows how engineered material inputs—chemistry and degradation, stiffness and geometry, coating and surface design, and cytokine release timing—engage the macrophage compartment through innate sensing. Macrophages adopt a spectrum of states: inflammatory (TNF-α, IL-1β, reactive oxygen species), reparative (IL-10, arginase-1), and pro-fibrotic (TGF-β, PDGF), with the metabolic state (glycolysis versus oxidative phosphorylation) further modulating the output. From above, adaptive immune inputs—Treg-derived IL-10 and TGF-β, TH2-derived IL-4 and IL-13, and B-cell complement activation—converge on the macrophage compartment to amplify or restrain these programs. In a traumatic muscle injury model, Sadtler and colleagues showed that ECM scaffolds induced a TH2/IL-4-associated immune program that was required for constructive remodeling and functional muscle recovery [32], and a broader comparison of biologic and synthetic materials confirmed that the immune program established around the material determines whether stromal remodeling proceeds constructively or towards fibrosis-prone remodeling [26].

The bidirectional arrows at the center of Figure 2 capture the coupled nature of the macrophage–fibroblast axis. Macrophage-derived TGF-β, PDGF, and IL-4 drive fibroblast recruitment, myofibroblast differentiation (αSMA, periostin, fibroblast activation protein), and ECM production (collagen I/III, fibronectin). In return, fibroblast-derived IL-6, CCL2, and CXCL12, together with matrix stiffening, sustain macrophage persistence and activation. In tunable PEG hydrogels, stiffer matrices led to more FAK-dependent remodeling, stronger αSMA-positive myofibroblast activation, higher collagen I deposition, and thicker fibrotic capsules; FAK inhibition reduced these fibrotic changes and altered the immune niche around the implant [14]. A second study showed that macrophages close to nondegradable polymer implants maintained a glycolytic metabolic program linked to inflammatory and fibroblast-activating signals [15]. Together, these findings show that FBR-associated fibrosis is shaped not only by which cells accumulate around the implant but also by the mechanical, immune, and metabolic conditions that keep the response from resolving.

The mechanical microenvironment (bottom right of Figure 2)—matrix stiffness, FAK signaling, and cyclic strain—provides additional input to the fibroblast compartment, reinforcing myofibroblast differentiation and pro-fibrotic ECM remodeling. Both cell compartments contribute to the ECM and capsule (collagen I/III deposition, matrix crosslinking, capsule maturation) and to the vasculature (angiogenesis, pericyte investment, perfusion). The integrated output of this system determines tissue fate: when immune activation resolves and stromal remodeling is coordinated, the outcome is constructive remodeling—immune resolution, organized ECM, stable vascularization, and implant integration; when macrophage persistence and fibroblast activation are mutually reinforcing and unresolved, the outcome is fibrotic encapsulation—FBGC formation, dense collagen capsule, avascular niche, and implant isolation.

Fibroblasts should therefore not be viewed merely as terminal effector cells of scar formation. They act as immune–stromal regulators that integrate cytokine, matrix, metabolic, and mechanical signals and convert them into tissue-level outcomes. Through crosstalk with macrophages, T cells, and matrix-derived cues, they influence leukocyte recruitment, ECM turnover, myofibroblast persistence, and the balance between resolution and chronic fibroinflammatory stabilization [5,6,19].

Adaptive immunity shapes fibroblast behavior and long-term implant fate through mechanisms that extend beyond the innate phase. Although the foreign body response literature has traditionally emphasized macrophages, T cells—particularly Tregs and TH2 cells—can influence fibroblast activation, biomaterial-associated fibrosis, and long-term tissue outcome. Importantly, type-2 immunity should not be interpreted as uniformly pro-regenerative. Transient spatially restricted IL-4/IL-13-associated responses can support constructive remodeling in some scaffold settings, whereas persistent type-2 signaling can sustain fibroblast activation, collagen deposition, and fibrotic remodeling. Likewise, TGF-β can contribute to immune regulation or repair in one context while promoting myofibroblast differentiation and fibrosis in another. These apparently contradictory observations emphasize that the signal timing, duration, tissue context, and the surrounding mechanical and cytokine environment are critical determinants of the outcome [2,4,6,18,23,32,33,34].

MSCs and MSC-derived EVs can influence immune responses around biomaterials. Depending on how they are prepared and delivered, they may reduce inflammatory signaling, alter macrophage behavior, and support Treg-associated responses [4,23,31,35]. Together, these findings suggest that cell-enabled approaches can shape the immune conditions under which remodeling occurs. Stromal remodeling is not simply a downstream consequence of inflammation: fibroblasts and matrix can also feed back on immune activity [6,19].

## 7. Fibroblast Heterogeneity and Stromal Immune Niches

Fibroblasts are not a uniform population, and αSMA expression alone does not describe the full range of stromal states involved in repair and fibrosis. For example, single-cell analysis of murine skin wounds resolved twelve fibroblast clusters, including sequential contractile states and apparently distinct lineages. Lineage-tracing experiments further identified a hematopoietic-derived subset contributing to wound myofibroblasts and rare regenerated adipocytes [36]. These findings illustrate why αSMA alone cannot identify fibroblast origin or distinguish among functionally different wound-associated stromal states. Single-cell and lineage-tracing studies have identified fibroblast populations that vary with tissue origin, anatomical location, injury stage, and remodeling outcome [36,37].

Some fibroblast states contribute to matrix organization, vascular support, and restoration of tissue structure, whereas persistent activation of contractile or matrix-producing states can promote fibrosis. A broader marker panel is therefore needed to distinguish myofibroblast differentiation from inflammatory, matrix-remodeling, or repair-associated fibroblast states [38].

Fibroblasts also interact with immune cells within spatially organized tissue niches. Macrophage-derived mediators can alter fibroblast activation, while fibroblast-derived cytokines, chemokines, and matrix-remodeling enzymes can influence immune-cell recruitment and behavior. Matrix deposition, degradation, and crosslinking further change local mechanics and immune signaling. These reciprocal interactions help explain why an early change in macrophage markers does not always predict the later stromal or tissue outcome.

## 8. Mitochondrial Dynamics and Organelle-Level Immune Regulation

The persistent glycolytic program identified in implant-proximal macrophages around nondegradable polymer implants indicates that chronic foreign body responses involve sustained immunometabolic reprogramming [15]. Mitochondrial fission and fusion, mitophagy, mitochondrial bioenergetics, and mitochondrial reactive oxygen species are established regulators of macrophage inflammatory signaling and functional state [39]. However, these organelle-level mechanisms have rarely been examined directly in macrophages at biomaterial interfaces.

Direct biomaterial evidence is beginning to emerge. An ion-releasing short fiber–microsphere scaffold was shown to restore macrophage mitochondrial metabolic homeostasis, reduce glycolysis through the downregulation of NF-κB p65 signaling, modulate macrophage inflammatory responses, promote osteogenic signaling in bone marrow stromal cells, and enhance bone regeneration in vivo [40]. This study supports positioning mitochondrial state as an emerging biological-process layer within the proposed framework, while also showing that the current evidence remains context-specific. Future studies should determine whether material stiffness, surface chemistry, degradation products, or controlled immune cues alter the mitochondrial morphology, respiratory function, or mitochondrial ROS in peri-implant macrophages and whether these changes predict fibroblast activation, matrix deposition, or fibrotic capsule formation.

## 9. Architecture and Mechanics as Immune Variables

A turning point in the field is the recognition that physical and architectural properties of biomaterials directly influence the immune response, rather than serving only as structural parameters. Stiffness, viscoelasticity, porosity, interconnectivity, roughness, hydrophilicity, geometric scale, and coating design can all influence which cells enter a niche, how long they remain there, how they are activated, and what kind of matrix is eventually produced [3,5,8,28]. Physical design should therefore not be treated as secondary to biochemical design. It is one of the main ways engineered materials shape immune–stromal behavior.

Geometry is one of the clearest examples. Larger alginate spheres generated much less foreign body fibrosis than smaller ones, with reduced immune-cell deposition and lower myofibroblast-associated encapsulation [41]. In a long-term subcutaneous implantation study, microporous annealed particle scaffolds produced minimal classical capsule formation and supported variable degrees of cell infiltration and matrix deposition. However, pore collapse and complement-associated changes were observed in most long-term samples, whereas complete infiltration and vascularization occurred in only one 12-month specimen [42]. In a separate in vitro study, precisely engineered 3D pore architectures altered macrophage responses to inflammatory stimulation without independently imposing a fixed polarization state [43]. Pore design can therefore shape how immune cells respond to a material even when it does not dictate phenotype by itself.

McWhorter and colleagues showed that enforced macrophage elongation on micropatterned substrates increased M2-associated marker expression without exogenous cytokines and that this effect depended on cytoskeletal organization and actomyosin contractility [30]. One-dimensional wrinkled topographies produced a related response, increasing arginase-1 and IL-10, reducing TNF-α, and altering collagen distribution around implanted materials [22]. Under moderate cyclic strain, hPBMC-seeded scaffolds showed a more immunoregulatory macrophage balance together with early collagen and sulfated glycosaminoglycan deposition. In an in vitro PCL scaffold system, the relative influence of architectural scale, orientation, and local stiffness on human macrophage morphology, polarization-associated gene expression, and TNF-α/IL-10 secretion depended on the dimensions of the structural cues [44].

Coating design adds another physical layer, because the host may respond not only to the bulk material but also to the thickness and surface properties of the interface it encounters. Yan and colleagues used an ultrasonic sprayable poly(acrylic acid)/polyethylenimine (PAA/PEI) polyelectrolyte coating on PDMS implants and showed that both the surface charge and coating thickness altered fibrotic capsule formation after subcutaneous implantation. Electronegative coatings produced the strongest anti-FBR effect, with lower near-interface collagen density and thinner capsules than neutral, positive, and uncoated surfaces. Thicker electronegative coatings reduced the capsule thickness more effectively than thinner coatings, consistent with the possibility that cells may sense the underlying stiff PDMS substrate when the soft coating is too thin [27].

These examples show that geometry, topography, stiffness, and coating architecture affect not only tissue ingrowth but also the immune conditions under which remodeling occurs [3,5,8,28]. In a tunable PEG hydrogel system, stiffer materials increased FAK-dependent myofibroblast activation, collagen deposition, and fibrotic capsule formation; FAK inhibition reduced these changes and also altered the immune niche around the implant [14]. Material architecture therefore does not simply support tissue repair after immune activation has already occurred. It participates directly in how the host interprets the implant and helps determine whether the response moves toward constructive remodeling or fibrotic encapsulation.

Similar pore sizes, stiffness ranges, or geometrical features may produce different in vivo outcomes because material chemistry, pore interconnectivity, degradation kinetics, animal species and strain, implantation site, and follow-up duration differ among studies. Differences in immune assays and outcome definitions add further variability. These physical cues should therefore be viewed as context-dependent design variables rather than universal predictors of constructive remodeling.

## 10. Immune-Microenvironment Engineering for Islet Encapsulation and Metabolic Tissue Applications

Pancreatic islet and stem cell-derived beta-cell encapsulation provides a clinically relevant case study for applying the principles of immune-microenvironment engineering, because controlling the host immune response is central to long-term graft function.

Islet transplantation can provide functional cell replacement for selected patients with type 1 diabetes, but long-term graft survival remains limited by immune-mediated rejection and fibrotic responses. Biomaterial encapsulation strategies, typically alginate-based microspheres or conformal coatings, aim to create an immune-protective barrier while permitting diffusion of oxygen, nutrients, and therapeutic insulin. However, encapsulated islets are avascular and have a high oxygen demand. The device thickness, cell-loading density, and vascularity at the implantation site can therefore produce local hypoxia, impair insulin secretion, and compromise graft survival. Fibrotic overgrowth further increases the diffusion distance and links the foreign body response directly to progressive loss of graft oxygenation [45]. These requirements create an inherent design tradeoff: increasing immune isolation may also restrict oxygen and nutrient transport. Oxygen-generating biomaterials have reduced hypoxic stress and preserved islet metabolic activity and glucose responsiveness in preclinical systems [46].

Veiseh and colleagues demonstrated that 1.5 mm alginate spheres elicited less immune-cell deposition and fibrosis than conventionally sized 0.5 mm spheres. When used to encapsulate rat pancreatic islets, the larger capsules maintained blood-glucose control for up to 180 days in diabetic mice [41]. Vegas and colleagues subsequently identified triazole-modified alginates that reduced macrophage recognition and fibrotic overgrowth in rodents and non-human primates [21]. However, the non-human-primate experiments in that study evaluated the foreign-body response to the materials without encapsulated islets and therefore did not assess graft survival or glycemic function. Using one of these lead chemistries, triazole–thiomorpholine dioxide (TMTD)-modified alginate, a companion functional study from the same group encapsulated human stem-cell-derived β cells in 1.5-mm spheres. These implants showed reduced immune-cell and fibrotic deposition and maintained glycemic correction and glucose responsiveness in immunocompetent diabetic C57BL/6J mice until graft retrieval at 174 days, without systemic immunosuppression [47]. Together, these studies distinguish material-level resistance to the foreign-body response from direct evidence of long-term graft function.

Related immunomodulatory platforms, including MSC-derived exosome-functionalized scaffolds [23] and hydrogel-supported MSC licensing [48], illustrate strategies for localized immune regulation. However, these approaches have not yet demonstrated efficacy around encapsulated beta cells in the cited studies. Direct evaluation in islet-specific models will therefore be required before their effects can be extended to stem cell-derived beta-cell encapsulation.

The clinical experience remains more limited than the preclinical literature suggests. In a small clinical study, an oxygen-refueled macroencapsulation device containing donor human islets produced detectable graft activity, but HbA1c levels and exogenous insulin requirements did not improve [49]. More recent trials using stem-cell-derived pancreatic products have demonstrated C-peptide production and improved glucose-control measures in a subset of recipients. However, these vascular-access devices required systemic immunosuppression, and the surviving β-cell mass remained limited [50].

These clinical findings support the feasibility of stem-cell-derived β-cell replacement but do not yet establish durable immunoisolating encapsulation. Although stem-cell-derived products may reduce dependence on donor islets, their performance remains constrained by oxygen and nutrient transport, vascular support, control of fibrotic overgrowth, and preservation of glucose-responsive insulin secretion. Current clinical evidence is insufficient to determine whether improvements in material-level foreign body response will consistently produce durable functional benefit.

## 11. Toward a Design Taxonomy

Across material classes and tissue contexts, the literature can be interrogated through four linked questions: What is engineered? Which biological process is engaged? What stromal response follows? What tissue-level outcome changes? Figure 3 organizes these questions into a four-layer taxonomy linking engineered inputs, biological processes, stromal responses, and tissue-level outcomes. Its purpose is not to rank studies but to reveal where experimental evidence directly connects these levels and where downstream links remain unmeasured. This distinction is important because a well-characterized early immune response does not necessarily predict stromal remodeling or long-term function.

The four layers are shown in sequence to make the framework easier to follow, but the biological response does not move in only one direction. Fibroblast-derived mediators can alter the immune-cell recruitment and state, while matrix deposition, degradation, and stiffening can change the immune mechanosensing and inflammatory signaling. These feedback processes may support resolution and integration or reinforce persistent inflammation and fibrosis. Although the framework is presented sequentially for design purposes, these relationships are not strictly unidirectional. Fibroblast-derived signals and changes in ECM composition and stiffness can feed back on macrophage activation and other immune processes, thereby modifying the subsequent remodeling trajectory.

The first layer (engineered inputs) is the most directly accessible experimentally. Studies have systematically varied controlled cytokine delivery [16,17,24], material chemistry, scaffold composition, and interface coatings [21,26,27,40], geometry, topography, and architecture [22,30,41,42,43,44], and stiffness-dependent mechanotransduction [14]. Together, these studies demonstrate that defined biochemical and physical variables can be manipulated and linked to measurable host responses.

The evidence for the second layer (biological processes engaged) is clearest for early innate mechanisms. Protein adsorption is an early determinant of host recognition, as demonstrated directly for fibrinogen-mediated inflammatory responses [20] and discussed in broader FBR frameworks [1,2,3,8]. Distinct downstream processes—including persistent macrophage glycolysis [15], mitochondrial metabolic regulation [40], cytoskeletal and topography-dependent macrophage responses [22,30,43,44], material-dependent myeloid programming [21,26,51], and macrophage–fibroblast crosstalk [14,15]—have also been documented. However, these mechanisms are not consistently connected to downstream stromal or functional outcomes within the same study. Adaptive immune mechanisms remain less well defined, particularly how specific material properties initiate Treg- and TH2-associated signaling or shape B-cell responses [32,33]. These unresolved links represent a significant evidence gap.

The third layer (stromal responses) is supported by a growing but uneven body of evidence. Direct examples include FAK-dependent myofibroblast activation and collagen I deposition in response to hydrogel stiffness [14], fibroblast-activating inflammatory output sustained by persistent macrophage glycolysis, vascular adaptation following sequential cytokine delivery [16], reduced capsule formation and improved integration after IL-4 delivery [17], reduced myofibroblast accumulation and collagen deposition around modified alginates [21], and contrasting connective-tissue remodeling around biological and synthetic scaffolds [26]. An ion-releasing short fiber–microsphere scaffold also linked macrophage mitochondrial metabolic regulation to enhanced osteogenic differentiation of BMSCs [40]. Other studies characterize macrophage responses to engineered cues without examining fibroblasts, ECM organization, or vascular remodeling [30,43,44], leaving the downstream stromal link unresolved. Fibroblast heterogeneity and the spatial organization of stromal remodeling across the implant–tissue interface also remain incompletely characterized.

The fourth layer (tissue-level outcomes) is the least consistently measured. Functional outcomes were demonstrated in selected studies, including enhanced skin-wound repair [23], prolonged glycemic control with encapsulated islets [41], and enhanced bone regeneration [40]. Other studies reported structural outcomes such as vascularization, implant integration, altered connective-tissue organization, or fibrotic capsule formation [14,16,17,21,22,26,27,42], but did not consistently assess organ-level or long-term functional performance. This incomplete connection between mechanism and function remains a central barrier to translation.

The taxonomy is most useful not as a static classification but as a design checklist: studies that examine multiple layers provide more complete evidence linking an engineered input to downstream biological, stromal, and tissue outcomes. However, cross-layer completeness does not by itself establish predictive validity or clinical translatability.

Table 1 maps selected primary studies onto the proposed four-layer framework. Studies were included to represent both complete and partial experimental links; “not evaluated in this study” identifies an unmeasured layer rather than an outcome inferred from the reported biological response.

**Table 1:** Selected primary studies mapped to the four-layer design framework.

Engineered Input	Biological Process	Stromal Response	Tissue Outcome	Citation
Triazole-modified alginate chemistry	Reduced macrophage recognition and macrophage/neutrophil recruitment	Reduced αSMA-positive myofibroblast coverage and collagen deposition	Material-only FBR assessment; reduced fibrotic overgrowth in rodents and non-human primates for up to 6 months in primates; functional islet transplantation was not evaluated	[21]
Alginate sphere geometry, large vs. small	Reduced immune-cell and cellular deposition on larger spheres	Reduced αSMA-positive cellular coverage and collagen-associated fibrotic overgrowth	Reduced fibrosis; encapsulated islets maintained glucose control for up to 180 days in diabetic mice	[41]
TMTD-modified alginate spheres encapsulating human stem-cell-derived β cells	Reduced association of macrophages, neutrophils, B cells, and CD8^+^ T cells without systemic immunosuppression	Reduced cellular deposition, αSMA-positive cellular coverage, and collagen-rich fibrotic overgrowth	Glycemic correction and glucose responsiveness maintained until graft retrieval at 174 days; viable insulin-producing cells remained in retrieved implants	[47]
Sequential IFN-γ/IL-4 delivery	Staged macrophage polarization in vitro	Increased CD31-positive vascularization, significant in the IFN-γ group	Enhanced scaffold vascularization; bone regeneration was not assessed	[16]
IL-4-eluting layer-by-layer coating on polypropylene mesh	Early shift toward M2-associated macrophage responses	Reduced capsule area and thickness; altered collagen organization	Reduced capsule thickness; improved tissue integration at 90 days	[17]
MSC-derived exosome-functionalized scaffold	Reparative macrophage, Treg, and TH2-associated responses	Improved collagen deposition; re-epithelialization and vascularization	Enhanced wound vascularization and repair	[23]
3D hydrogel-supported MSC licensing	Increased immunoregulatory gene expression and T-cell suppression	Not directly assessed in vivo	Not directly assessed; in vitro study	[48]
Tunable PEG hydrogel stiffness	FAK-dependent mechanotransduction and immune remodeling	Myofibroblast activation and collagen I deposition	Stiffness-dependent fibrotic capsule formation; reduced by FAK inhibition	[14]
Nondegradable polymer implant	Persistent glycolytic macrophage programming at the implant interface	Profibrotic signaling, myofibroblast association, and ECM deposition	Persistent peri-implant inflammation and fibrosis	[15]
1D wrinkled topography	Macrophage shape-sensing; increased arginase-1 and IL-10; reduced TNF-α	Altered collagen organization	Modified early host response at 7 days; long-term integration was not assessed	[22]
Moderate cyclic strain	Strain-dependent macrophage modulation	Early collagen and sulfated glycosaminoglycan deposition	Not directly assessed; in vitro study	[51]
Precisely engineered 3D pore architecture	Pore size altered macrophage responses to inflammatory stimulation without independently imposing a fixed polarization state	Not evaluated in this study	Not evaluated; in vitro macrophage study	[43]
PAA/PEI coating with variable charge and thickness	Not evaluated in this study	Reduced peri-implant collagen density and capsule thickness	Thinner fibrotic capsule; strongest effect with thicker electronegative coatings	[27]
Biologic ECM scaffold vs. synthetic PE/PEG scaffold	Divergent innate immune programs; type-2/pro-regenerative response vs. neutrophilic/FBR response	Cellular infiltration and remodeling with ECM vs. αSMA-rich collagenous encapsulation with synthetic materials	Regenerative structural remodeling vs. fibrosis-prone encapsulation; functional muscle recovery was not assessed	[26]
Microporous annealed particle scaffold, pore architecture	Sustained immune-cell infiltration without classical capsule-forming response	Tissue infiltration; matrix deposition without dense capsule	Variable long-term integration; complete vascularization occurred in one 12-month sample	[42]
Biomimetic macrophage–fibroblast co-culture fibrosis assay	Material-dependent macrophage–fibroblast interaction; in vitro modeling of fibrotic output	Material-dependent collagen remodeling and TGF-β1 production	Candidate in vitro fibrosis assay; in vivo predictive validity remains untested	[52]
Ion-releasing short fiber–microsphere scaffold (FM@CeZnHAP)	Restoration of macrophage mitochondrial metabolic homeostasis; reduced glycolysis and mitochondrial oxidative stress; modulation of NF-κB p65 signaling and macrophage inflammatory balance	Enhanced osteogenic differentiation of BMSCs and accelerated ossification-center formation	Reduced early inflammation and enhanced bone regeneration in rat femoral condylar defects	[40]

Studies are organized by engineered input, biological process, stromal response, and tissue-level outcome. Not all studies evaluated every layer; “not evaluated in this study” denotes an experimentally unmeasured link rather than an inferred outcome.

## 12. Translational Gaps and the Need for Standardization

Despite conceptual progress, outcome assessment in the field remains fragmented. Many studies report early cytokines or limited macrophage marker panels without linking those results to fibroblast state, ECM organization, vascular maturation, foreign body giant cell formation, capsule structure, or functional tissue outcome [4,19,53].

Most mechanistic evidence in this field comes from in vitro experiments and small-animal implantation models. Large-animal and clinical evidence remains limited, and changes in cytokines, immune-cell markers, or capsule histology do not necessarily predict durable human function. Clinical studies also differ in material composition, implantation site, patient population, follow-up, and outcome definition, which prevents standardized quantitative comparison.

Heterogeneity is also substantial within the preclinical literature. The studies differ in material chemistry, architecture, degradation, animal species and strain, implantation site, injury context, follow-up duration, and immune or stromal assays. Apparently conflicting results may therefore reflect differences in experimental context rather than the effect of a single material variable. Findings from one material or model should not be treated as universally transferable.

As a result, it is often unclear whether an intervention has truly changed tissue fate or has only shifted an early inflammatory profile. Four specific and actionable changes would substantially improve the field.

First, studies should adopt multiparameter stromal profiling panels that capture the full spectrum of peri-implant tissue remodeling. Fibroblast activation should be assessed using a combination of markers including αSMA, periostin, and fibroblast activation protein (FAP), which together distinguish myofibroblast differentiation from other activated fibroblast states. ECM composition should be characterized beyond the total collagen content to include the collagen I/III ratio and fibronectin deposition, which reflect the maturity and mechanical character of the fibrotic response. Vascular remodeling should be assessed using CD31 and αSMA co-staining to distinguish immature angiogenic sprouts from mature, pericyte-invested vessels.

Second, macrophage profiling should move beyond M1/M2 binary classification toward high-dimensional approaches that capture the full range of biomaterial-induced myeloid states. Mass cytometry (CyTOF) and single-cell RNA sequencing (scRNA-seq) have already revealed that tissue macrophages occupy a continuous spectrum of activation states that do not map cleanly onto M1 or M2 categories [18,29]. Applying these tools to biomaterial-associated macrophage populations would allow the field to define which specific myeloid states are associated with constructive remodeling versus fibrotic encapsulation.

Third, intermediate mechanistic models—both computational and in vitro co-culture systems—should be used alongside in vivo studies rather than treated as preliminary or supplementary. One computational framework has already linked material properties, macrophage–fibroblast interactions, stiffness, and ECM accumulation in a model of implant-induced fibrosis [54], and a biomimetic in vitro co-culture assay has connected material type with fibroblast–macrophage fibrotic output [52]. These approaches do not replace in vivo validation, but they can generate mechanistic predictions and provide experimentally tractable fibrosis assays that are faster and more controllable than animal studies.

Fourth, longitudinal outcome assessment with functional endpoints should be prioritized rather than treated as optional. Studies should incorporate appropriately long-term follow-up, often extending to 90 days or beyond for subcutaneous implants and longer for load-bearing or organ-integrated devices, depending on the material and application, and should report functional endpoints—mechanical performance, perfusion, organ function, or tissue architecture—alongside histological and molecular readouts. The field would also benefit from stronger adaptive immune profiling, as many biomaterial studies still do not examine T-cell or B-cell contributions to long-term stromal remodeling and implant outcomes [6,19,33].

The framework will require prospective testing to determine whether early immune and stromal measurements predict later tissue function across different materials, implantation sites, species, and clinical settings.

## 13. Conclusions

Immune-microenvironment engineering in biomaterials for tissue repair and regeneration is ultimately about shaping how early host responses lead to later stromal remodeling and tissue outcome. The reviewed studies point to a central design principle: repair depends on the timing, localization, resolution, and stromal consequences of immune activation.

There is no single design rule that consistently drives repair across all biomaterial systems. Instead, the outcome tends to depend on a recurring set of factors, including timing, signal persistence, architecture, mechanics, interface design, immune modulation, macrophage–fibroblast crosstalk, metabolic state, and mechanotransductive feedback. Together, these factors influence whether a material supports constructive remodeling and stable integration or shifts toward persistent inflammation and fibrosis.

Biomaterial outcomes are therefore best understood as the result of linked immune-stromal programs that unfold over time. The challenge is to control how engineered inputs shape host sensing, stromal organization, and tissue fate. A more predictive field will require studies that connect material properties, biological mechanisms, stromal responses, and long-term tissue outcomes within the same experimental setting. The next step for the field is therefore not only to identify anti-inflammatory materials but also to define which engineered cues can reproducibly coordinate immune activation, stromal remodeling, vascular adaptation, and long-term integration.

Used as an experimental checklist, the framework supports measuring material properties and degradation; innate and adaptive immune-cell composition and state; fibroblast activation and heterogeneity; extracellular matrix organization and vascular maturation; and long-term function relevant to the intended application. These measurements should be collected at more than one time point, because no single macrophage or fibroblast marker can distinguish transient repair-associated activation from persistent fibrotic remodeling.

Prospective validation is needed to determine whether the framework improves experimental comparability or whether early immune–stromal patterns predict later function across materials, implantation sites, species, and clinical settings. Until then, its main value is to identify the missing links between material design, biological mechanism, stromal response, and tissue function and to guide more complete experimental assessment.

## Figures and Tables

**Figure 1:**
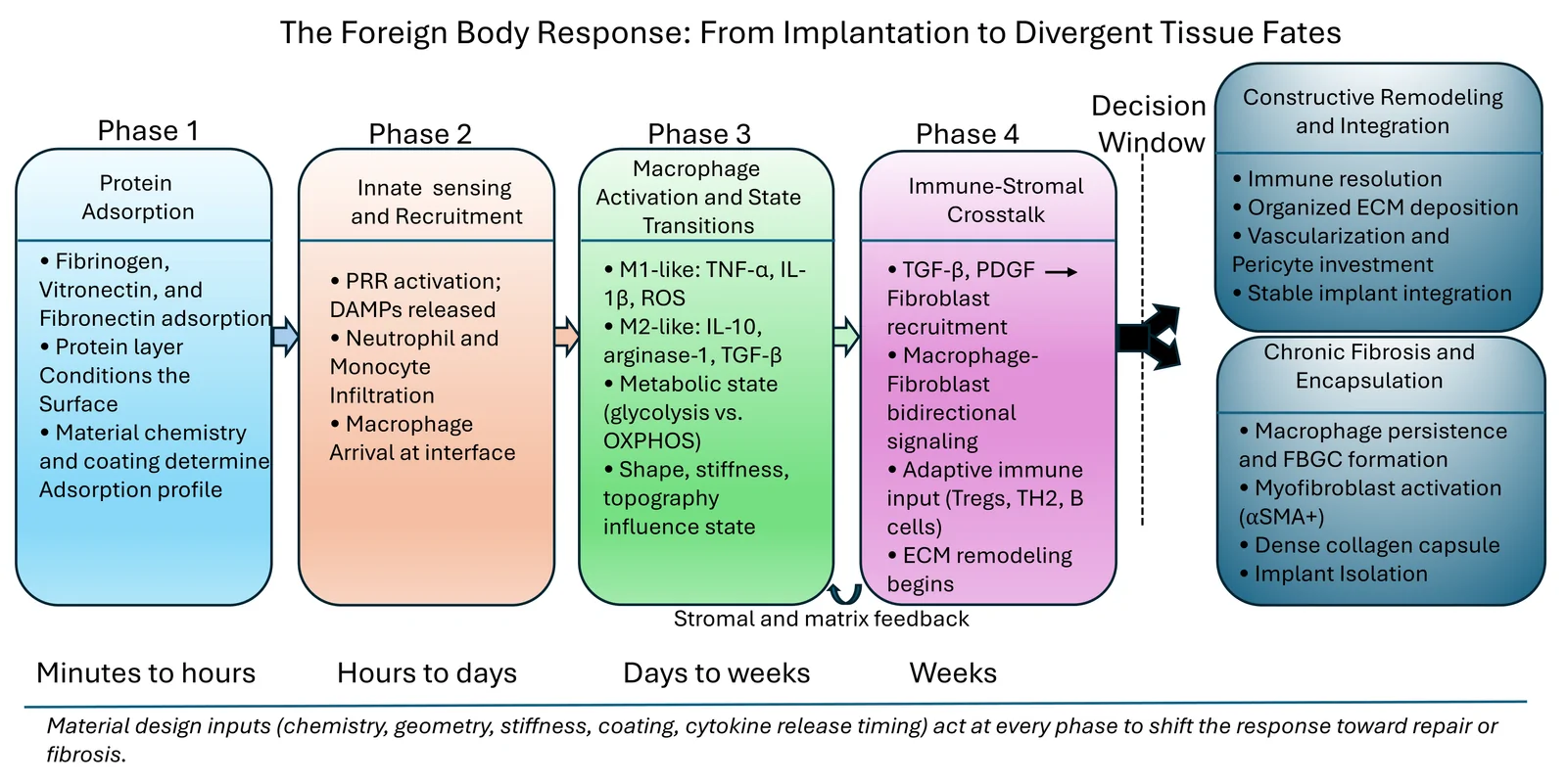
The foreign body response as a sequential designable process leading to divergent tissue fates. The FBR unfolds through four temporally overlapping phases, protein adsorption (minutes to hours), innate sensing and recruitment (hours to days), macrophage activation and state transitions (days to weeks), and immune–stromal crosstalk (weeks), before diverging across a conceptual Design Decision Window toward either constructive remodeling and integration or chronic fibrosis and encapsulation. Material design inputs including chemistry, geometry, stiffness, coating, and cytokine release timing act at every phase to shift the trajectory of the response. The Design Decision Window (dashed line) is a conceptual construct indicating a period during which the response may remain redirectable by engineered intervention; as fibrosis becomes established, stabilization can become increasingly self-reinforcing. M1-like and M2-like labels are used as simplified marker programs rather than fixed macrophage phenotypes. PRR, pattern-recognition receptor; DAMP, damage-associated molecular pattern; TNF-α, tumor necrosis factor-α; TGF-β, transforming growth factor-β; PDGF, platelet-derived growth factor; ECM, extracellular matrix; αSMA, alpha-smooth muscle actin; FBGC, foreign body giant cell; OXPHOS, oxidative phosphorylation. Backward arrows indicate feedback from fibroblast-derived mediators, extracellular matrix remodeling, and changes in local tissue mechanics.

**Figure 2:**
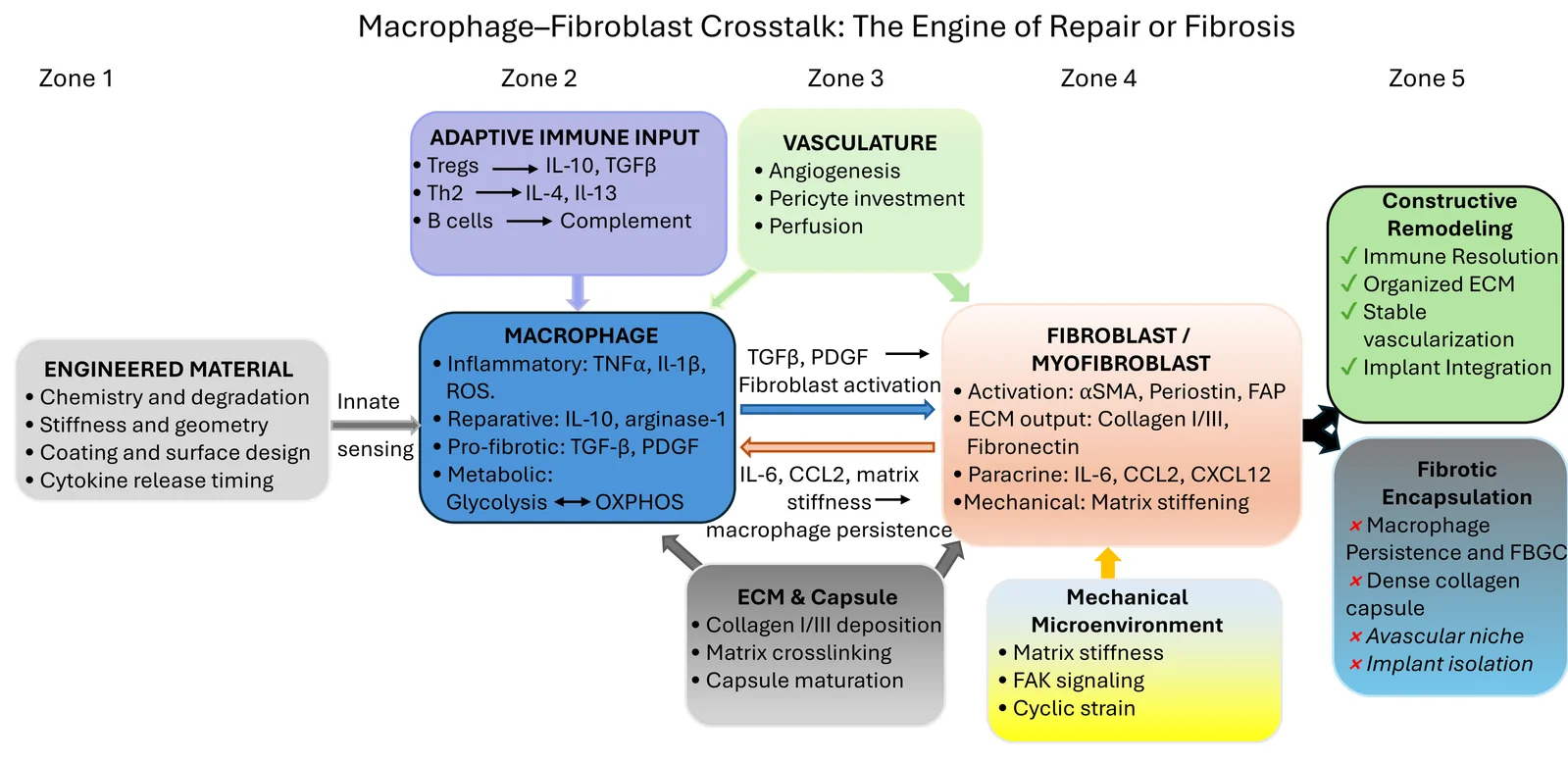
The macrophage–fibroblast axis as a coupled immune–stromal system determining repair or fibrotic encapsulation. Engineered material inputs (left) engage the macrophage compartment through innate sensing, driving a spectrum of inflammatory, reparative, and pro-fibrotic activation states modulated by metabolic programming (glycolysis versus oxidative phosphorylation). Adaptive immune inputs—Treg-derived IL-10 and TGF-β, TH2-derived IL-4 and IL-13, and B-cell complement activation—converge on the macrophage from above. Bidirectional arrows between the macrophage and fibroblast/myofibroblast compartments indicate the coupled nature of this axis: macrophage-derived TGF-β and PDGF drive fibroblast activation and ECM production, while fibroblast-derived IL-6, CCL2, and matrix stiffening sustain macrophage persistence. The mechanical microenvironment (matrix stiffness, FAK signaling, cyclic strain) provides additional input to the fibroblast compartment. Both compartments contribute to ECM and capsule maturation and to vascular remodeling. The integrated output determines tissue fate: constructive remodeling (green, right) or fibrotic encapsulation (red, right). TGF-β is shown as a context-dependent signal that may contribute to immune regulation, repair, or fibrosis depending on timing and local tissue state. TGF-β, transforming growth factor-β; PDGF, platelet-derived growth factor; FAK, focal adhesion kinase; αSMA, alpha-smooth muscle actin; FAP, fibroblast activation protein; ECM, extracellular matrix; FBGC, foreign body giant cell; OXPHOS, oxidative phosphorylation.

**Figure 3:**
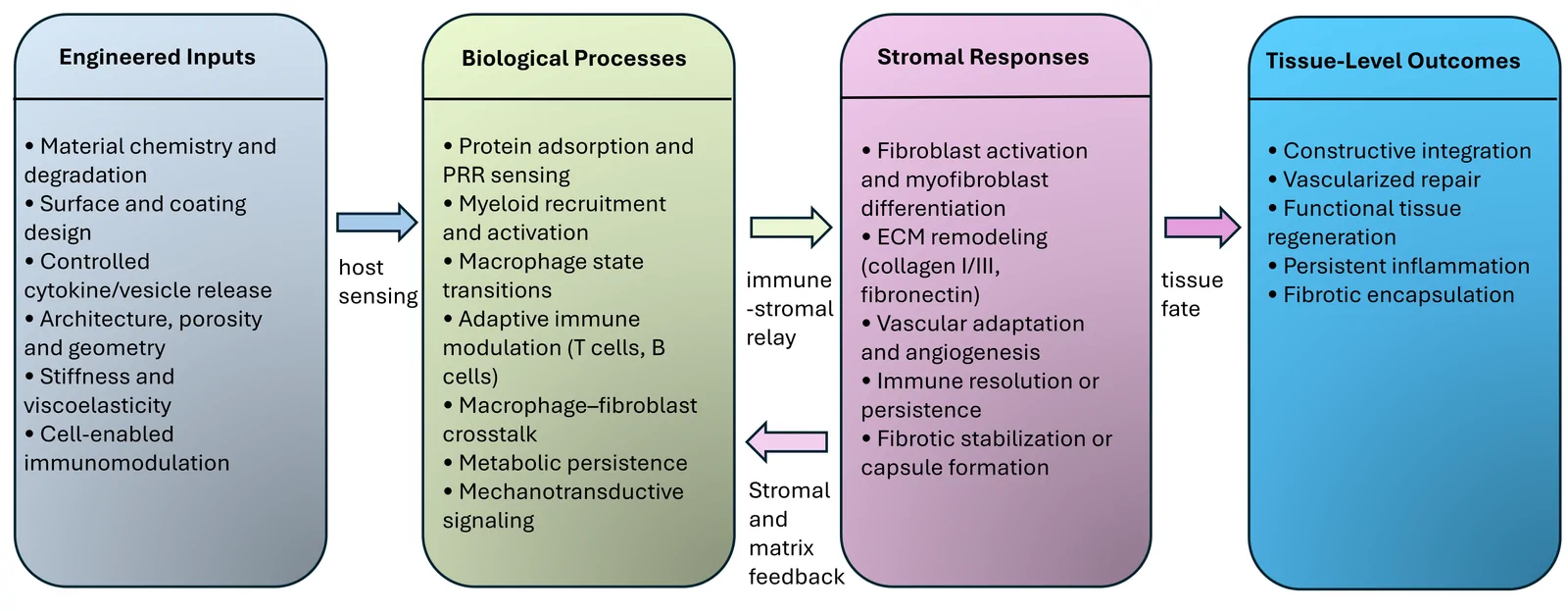
Four-layer design framework linking engineered material inputs to tissue-level outcomes through biological processes and stromal responses. Engineered inputs (left)—including material chemistry and degradation, surface and coating design, controlled cytokine or vesicle release, architecture and geometry, stiffness and viscoelasticity, and cell-enabled immunomodulation—engage biological processes (center-left) through host sensing and innate recognition. These processes shape stromal responses (center-right), including fibroblast activation, ECM remodeling, vascular adaptation, and immune resolution or fibrotic stabilization, which in turn determine tissue-level outcomes (right) ranging from constructive integration and vascularized repair to persistent inflammation and fibrotic encapsulation. The framework is intended as a design checklist: studies that examine multiple layers provide more complete evidence linking an engineered input to downstream biological, stromal, and tissue outcomes. However, cross-layer completeness does not by itself establish predictive validity or clinical translatability. Backward arrows indicate feedback from fibroblast-derived mediators, extracellular matrix remodeling, and changes in local tissue mechanics.

## Data Availability

No new data were created or analyzed in this study. Data sharing is not applicable to this article.
